# Editorial: Tailored Modulation of Interactions Between Biomolecules: Fundamentals and Applications

**DOI:** 10.3389/fmolb.2022.961452

**Published:** 2022-07-14

**Authors:** Matteo Ardini, Paola Baiocco, Adele Di Matteo, Giorgio Giardina, Adriana Erica Miele

**Affiliations:** ^1^ Department of Public Health, Life and Environmental Sciences, University of L’Aquila, L’Aquila, Italy; ^2^ Department of Biochemical Sciences, Sapienza University of Rome, Rome, Italy; ^3^ CNR Institute of Molecular Biology and Pathology, Roma, Italy; ^4^ Institut des Sciences Analytiques, UMR 5280 CNRS UCBL, University of Lyon, Villeurbanne, France

**Keywords:** biomolecules, surfaces, interactions, tailored modulations, nanostructures, biosensing

The interactions between biomolecules often require flexible surfaces to perform many different tasks such as recognition, regulation and signaling. These interactions rely on sophisticated, dynamic molecular mechanisms that must be tightly controlled within very crowded environments, such as cellular compartments, to allow tailored and tuned complexes, *e.g.*, receptor- and enzyme-ligand or chaperone-client. Fundamental studies ceaselessly reveal how biomolecules interact with each other to provide cells with structural and enzymatic pivotal characteristics. The term “interactome” firstly introduced in Sanchez et al. (1999) gives the idea of how the complexity of organisms can be, at least in part, explained by considering the multiple interactions between its own biomolecules, *e.g.*, proteins, DNA, lipids, small ligands and ions, rather than only its genetic content and organization. Dissecting these interactions is crucial to understand not only cellular functions, but also the molecular basis of diseases and designing new therapies as well as to create new bio-based approaches to build-up functional, responsive materials at the nano- and microscale, as summarized for instance by Isaacson and Díaz-Moreno (2019) and by Andreani et al. (2022).

Many representative examples about how biomolecular interactions drive cells’ life can be recalled. It is now clear, for instance, that multivalent interactions may trigger the assembly of the highly dynamic and reversible membrane-less organelles via liquid-liquid phase separation or LLPS as shown by Zhao and Zhang (2020): these organelles represent physicochemical and thermodynamic islands where proteins are present at very high concentrations, thus expanding their conformational and functional potential. Moreover, Wang et al. (2021) showed that an increasing set of proteins that physiologically undergo LLPS are also found in pathological aggregates that have been correlated to various diseases such as cancer, neurodegenerative and infectious diseases. Likewise, as highlighted by Liu et al. (2022) the biomolecular corona, *i.e.*, the layer of biomolecules, mainly proteins, adsorbing to the surfaces of nanoparticles (NPs), must be considered, and addressed as an essential component of nanomedicine designs and it is paramount to achieve a better understanding of the interaction between NPs and the specific biological environment they are exposed to.

The importance of understating the molecular interactions between biomolecules is reflected by the explosive trend of several tools and techniques occurred recently. The last years, for instance, have seen the breakthrough of the deep learning neural network architecture-based methods for protein structure prediction (AlphaFold2) developed by Jumper et al. (2021) and very recently also for the prediction of the structures of protein complexes with AlphaFold-Multimer. Recent studies showed that AlphaFold2 is not just a tool for modeling monomeric structures but can also model protein complexes. However, accurate prediction of protein-ligands interaction remains an epochal challenge. Furthermore, cryogenic microscopy (Cryo-EM) marked a step forward to investigate and disclose those interactions between biological molecules and their partners and ligands in fundamental studies as well as for biopharmaceutical purposes in the development of new drugs as marked by de Oliveira et al. (2021), Wigge et al. (2020). This technique is meant to revolutionize by getting rid over the limitations that affect traditional methods in structural biological, for instance poor solubility of the target(s) as in the case of membrane proteins or lipophilic ligands and the need of crystallization procedures, which freeze single target conformations.

This Research Topic pointed out special emphasis on the characterization of the interactions between molecular partners forming complexes, be it fully biological of hybrid. The contributions received are briefly described herein.

In the original article by Dubackic et al. the authors investigate the properties of α-Synuclein fibrils to aggregate into Lewis bodies by small angle neutron scattering (SANS). Lewis bodies are strongly associated with Parkinson’s and several other neurodegenerative diseases. The study examines α-Synuclein’s clusters formed under mild acidic conditions and with a wide range of different lipids mimicking different membrane systems. The main outcome of this work is that the clusters can be described as mass fractal aggregates whose dimensions may be reproduced using a simple model of rigid-rod clusters.

The review by La Manna et al. focuses on the role of SOCS proteins (Suppressor Of Cytokine Signaling) as putative source of protein-protein interaction inhibitors (PPI) of the signaling pathway JAK-STAT (JAnus tyrosine Kinase—Signal Transducers and Activators of Transcription). In particular, they show the power of peptido-mimetic compounds in modulating JAK-STAT interactions in inflammatory diseases and in cancer onset. In both cases peptides derived from specific SOCS are able in cellular models to down-regulate the pathway and hence revert to a physiological state. Therefore, tailor-made peptides might be designed and used to specifically target only some subsets of isoforms in order to avoid general side effects.

The original research paper by De Lauro et al. presents a software written to optimize therapeutic antibodies (Ab) by designing complementary interfaces with the antigen (Ag) to be neutralized. As a proof of concept, they applied the design of tailored Ab-Ag interfaces to SARS-CoV2 spike protein. In particular, they have taken into account specific Ag with and without glycosylation and Ag which were not affected by known mutations at the time of the research. They compare their results with known protein-protein docking softwares and discuss their outcomes.

When reporting on biological interactions and their use for practical purposes, bionanotechnology is currently being considered a flourishing area. Biomolecules, especially those forming supramolecular complexes, exhibit chemical features which are really wanted when building materials: they can self-assemble *via* non-covalent interactions and interact via nanomaterials in such a way to drive their controlled aggregation into new, hybrid and responsive structures. In this context, the review proposed by Passaretti reports on many examples where biomolecules such as DNA and proteins or even whole organisms such as viruses are used to assemble graphene and graphene oxide materials with geometrical architecture including fibers, sponges and films which are found beneficial in many areas, *e.g.*, energy storage, sensors, catalysis, air and water filtering as well as tissue engineering.

Due to the growing interest in the last few years towards materials based on biological building-blocks for many applications, such as plasmon-enhanced next-generation sequencing, biomolecular detection and biosensing, this Topic aims to provide new ideas for both fundamental and applied science at a molecular level.
